# Peritoneal carcinomatosis: patients selection, perioperative complications and quality of life related to cytoreductive surgery and hyperthermic intraperitoneal chemotherapy

**DOI:** 10.1186/1477-7819-7-5

**Published:** 2009-01-08

**Authors:** Gabriel Glockzin, Hans J Schlitt, Pompiliu Piso

**Affiliations:** 1 Department of Surgery, University of Regensburg Medical Center, Regensburg, Germany

## Abstract

**Background:**

Peritoneal tumor dissemination arising from colorectal cancer, appendiceal cancer, gastric cancer, gynecologic malignancies or peritoneal mesothelioma is a common sign of advanced tumor stage or disease recurrence and mostly associated with poor prognosis.

**Methods and results:**

In the present review article preoperative workup, surgical technique, postoperative morbidity and mortality rates, oncological outcome and quality of life after CRS and HIPEC are reported regarding the different tumor entities.

**Conclusion:**

Cytoreductive surgery (CRS) and hyperthermic intraperitoneal chemotherapy (HIPEC) provide a promising combined treatment strategy for selected patients with peritoneal carcinomatosis that can improve patient survival and quality of life. The extent of intraperitoneal tumor dissemination and the completeness of cytoreduction are the leading predictors of postoperative patient outcome. Thus, consistent preoperative diagnostics and patient selection are crucial to obtain a complete macroscopic cytoreduction (CCR-0/1).

## Background

Peritoneal carcinomatosis is a common sign of advanced tumor stage, disease progression or recurrence in numerous tumor entities of gastrointestinal or gynecological origin. Moreover, there are primary peritoneal malignancies such as malignant peritoneal mesothelioma or primary peritoneal carcinoma. In general, the diagnosis of peritoneal tumor manifestation is associated with poor prognosis. In the European multicenter EVOCAPE I study the median survival rates were 5.2 months for advanced colorectal cancer (CRC, n = 118) and 3.1 months for advanced gastric cancer (GC, n = 125), respectively[1]. The median survival rate in patients with stage IV ovarian cancer (OC) range from 12 to 23 months [2-4]. For diffuse malignant peritoneal mesothelioma (DMPM) median survival rates of less than one year are reported in most existing studies [5-7]. However, in a Phase II trial with systemic application of permetrexed and gemcitabine the median survival rate was 26.8 months in patients with malignant peritoneal mesothelioma [8]. The treatment of choice for patients with peritoneal surface malignancies is palliative systemic chemotherapy. In the past, surgery was performed in palliative intention for prevention or therapy of tumor-related complications such as gastrointestinal obstruction, bleeding or tumor perforation [9]. Solely, in ovarian cancer cytoreductive surgery was already established as an inherent part of the standard treatment regimen [10]. In the early 1990's Sugarbaker et al. introduced cytoreductive surgery (CRS) and hyperthermic intraperitoneal chemotherapy (HIPEC) as a new innovative therapeutic option for selected patients with peritoneal carcinomatosis [11,12]. Over the years peritoneal carcinomatosis treatment centers were established in the United States, Europe and Japan. Feasibility, efficacy and safety of CRS and HIPEC have been proved in numerous clinical trials. In the present review article patient selection, treatment strategy, mortality and morbidity rates and oncological outcome is reported regarding the different tumor entities.

### Cytoreductive surgery

**Figure 1:**
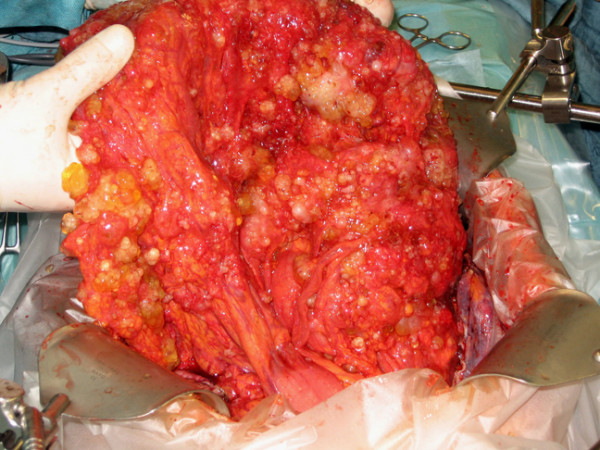
**'Omental cake' in a patient with peritoneal carcinomatosis arising from appendiceal cancer**.

**Figure 2:**
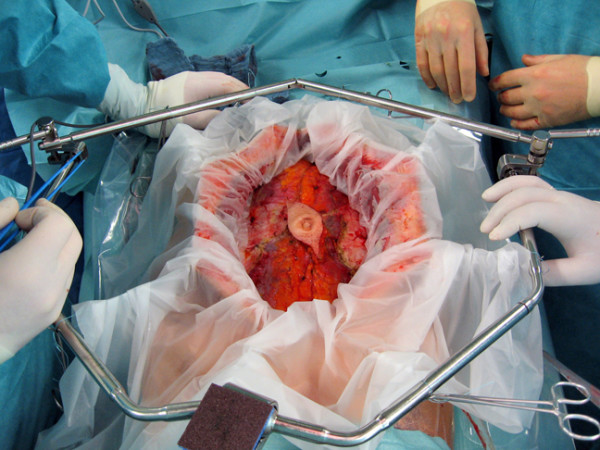
**Omphalectomy in a patient with umbilical tumor infiltration**.

CRS consists of numerous surgical procedures depending on the extent of peritoneal tumor manifestation. In appendiceal malignancies, the omental cake, a disseminated tumor infiltration of the greater omentum, represents the most affected abdominal area (Fig. 1). Surgery may include parietal and visceral peritonectomy, greater omentectomy, splenectomy, cholecystectomy, resection of liver capsule, small bowel resection, colonic and rectal resection, (subtotal) gastrectomy, lesser omentectomy, pancreatic resection, hysterectomy, ovariectomy and urine bladder resection. In patients with mucinous tumors and infiltration of the umbilicus, an omphalectomy is necessary. Extraperitoneal dissection may enable the anterior parietal peritonectomy and avoid a tumor contamination of the abdominal wall (Fig. 2). The extent of intraperitoneal tumor manifestation is determined using the peritoneal cancer index (PCI), a combined numerical score of lesion size (LS-0 to LS-3) and tumor localization (region 0–12) [13,14]. The aim of CRS is to obtain complete macroscopic cytoreduction (CCR-0/1) as a precondition for the application of HIPEC. The residual disease is classified intraoperatively using the completeness of cytoreduction (CCR) score. CCR-0 indicates no visible residual tumor and CCR-1 residual tumor nodules ≤ 2.5 mm. CCR-2 and CCR-3 indicate residual tumor nodules between 2.5 mm and 2.5 cm and > 2.5 cm, respectively [14].

### Hyperthermic intraperitoneal chemotherapy

In case of complete macroscopic cytoreduction (CCR-0/1) CRS is followed by hyperthermic intraperitoneal chemotherapy (HIPEC). The theoretical advantage of the intraperitoneal distribution of cytostatics is a high local concentration of the used agents and reduced systemic toxicity. *In vitro *studies could show that hyperthermia may potentiate the cytostatic effects. For example an improved tissue penetration could be shown for cisplatin. Moreover, hyperthermia leads to direct cytotoxic effects such as protein denaturation, induction of apoptosis and inhibition of angiogenesis [15].

**Figure 3:**
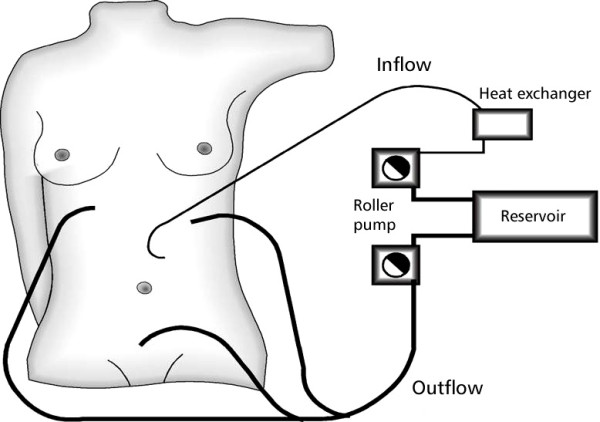
**Schematic diagram of HIPEC procedure**.

For the performance of HIPEC one inflow and three outflow drainages are placed subphrenically and in the small pelvis. The cytostatic agent is applied via the inflow drainage using a roller pump and heat exchanger in a closed system that allows perfusate circulation (Fig. 3). The intraperitoneal temperature is monitored by two sensors placed in the inflow catheter and in the Douglas pouch. The intraperitoneal temperature should reach 41–42°C leading to an inflow temperature of about 43°C.

Until today the cytostatic agents, combinations and concentrations used for HIPEC are not standardized for all peritoneal carcinomatosis centers worldwide. Thus, numerous different protocols are used for the different tumor entities. The perfusion times ranges from 30 to 120 minutes depending on the protocol and the drug used. Moreover, numerous different drugs and drug combinations are used (Table 1). HIPEC can be performed in open or closed abdomen technique. One of the leading advantages of the open technique is a better control of the intraperitoneal circulation and uniform distribution of the cytostatic agents. An important disadvantage is the increased risk of contamination compared to the closed abdomen technique. Although a comparism of the existing studies is difficult there seem to be no significant differences between the two techniques regarding morbidity and mortality rates as well as patient survival [16].

**Table 1:** Selected studies with CRS and HIPEC in patients with peritoneal carcinomatosis of different origin.

**Author, year**	**n**	**Tumor entity**	**Cytostatic agent(s)**	**Morbidity**	**Mortality**	**Median survival**	**Overall survival**	**Survival** **CCR-0/1**
				
				[%]	[%]	[months]	[%]	[%]
Verwaal, 2003[25,41]	105	CRC	MMC	35	8	22	28 (3-y)	45 (5-y)

Glehen, 2004[33]	506	CRC	MMC/LOHP	23	4	19	39 (3-y)	47 (3-y)

Shen, 2004[34]	77	CRC	MMC	30	12	16	25 (3-y)	44 (3-y)

Glehen, 2004[29]	49	GC	MMC	27	4	10	8 (5-y)	29 (5-y)

Hall, 2004[45]	34	GC	MMC	35	0	11	-	21 (5-y)

Yonemura, 2005[30]	105	GC	MMC/DDP	22	3	19	7 (5-y)	27 (5-y)

Feldmann, 2003[35]	49	DMPM	DDP	25	0	92	59 (5-y)	-

Deraco, 2006[36]	49	DMPM	DDP/DXR DDP/MMC	27	0	-	57 (5-y)	-

Yan, 2007[27]	70	DMPM	DDP/DXR	36	3	59	49 (5-y)	-

Piso, 2004[37]	19	OC	DDP; MITO	28	5	33	15 (5-y)	44 (5-y)

Cotte, 2007[38]	81	OC	DDP	14	3	28	-	-

Di Giorgio, 2008[26]	47	OC	DDP	21	4	30	17 (5-y)	26 (5-y)

CRC: colorectal cancer, GC: gastric cancer, PMP: pseudomyxoma peritonei, OC: ovarian cancer, DMPM: diffuse malignant peritoneal mesothelioma, MMC: mitomycin C, DDP: cisplatin, LOHP: oxaliplatin, DXR: doxorubicin, MITO: mitoxantrone

### Preoperative diagnostics and patient selection

Preoperative patient selection plays a pivotal role for the success of CRS and HIPEC regarding clinical as well as oncological patient outcome. Thus, preoperative diagnostics including physical examination, laboratory parameters, tumor markers (CA19-9, CEA, CA125, CA72-4), computed tomography of the chest, abdomen and pelvis with intravenous and oral/rectal contrast and endoscopy with or without endoluminal ultrasonography (colorectal and gastric cancer) are indispensable (Table 2). In some cases additional ultrasound, abdominal magnetic resonance imaging (MRI) and/or PET-CT may be helpful depending on the primary tumor and tumor dissemination [17]. However, Esquivel et al. have shown that preoperative CT-PCI does not correlate with the intraoperative PCI. In 52 patients with peritoneal carcinomatosis of colonic origin from 19 international centers the mean CT-PCI was 8.6 vs. 13.2 (Esquivel, SSO 2008). In our experience a leading reason for incomplete macroscopic cytoreduction is the intraoperative finding of disseminated tumor spots in the small bowel region. Thus, staging laparoscopy should be performed if necessary to determine tumor dissemination especially in patients with peritoneal carcinomatosis from gastric cancer but not in patients with DMPM because of the high risk of port side metastasis [18,19]. Anyway, the tumor entity should be taken into account. Whereas for example patients with peritoneal carcinomatosis of colonic origin with a PCI ≤ 20 qualify for CRS and HIPEC, the PCI in patients with gastric cancer should be < 10 or ≤15 [20,21]. In patients with pseudomyxoma peritonei arising from mucinous neoplasms PCI > 20 is no absolute exclusion criteria. In these patients tumor grading, extent of mesenteric invasion, liver metastasis and age play an important role in conjunction with PCI [22]. The Peritoneal Surface Malignancy Group defined eight clinical and radiological variables that increase the probability of complete macroscopic cytoreduction in patients with peritoneal carcinomatosis of colonic origin: (1) ECOG performance status ≤ 2, (2) no evidence of extra-abdominal disease, (3) up to three small, resectable parenchymal hepatic metastases, (4) no evidence of bilary obstruction, (5) no evidence of ureteral obstruction, (6) no evidence of intestinal obstruction at more than one site, (7) small bowel involvement: no evidence of gross disease in the mesentery with several segmental sites of partial obstruction and (8) small volume disease in gastro-hepatic ligament [20,23]. In patients with DMPM extra-abdominal and hepatic metastasis, histology, nuclear grade and mitotic count are crucial prognostic factors for preoperative patient selection and oncological outcome [18]. Most experts exclude patients with distant metastasis from primary and recurrent gastric cancer [21]. The ovary consensus panel (OCP) found no absolute contraindications for CRS and HIPEC in patients with ovarian cancer regarding tumor dissemination or metastasis. The access should be individually evaluated. Nevertheless, heart failure and pulmonary compromise preclude the combined treatment concept [24].

**Table 2:** Preoperative diagnostic workup.

**Essential preoperative diagnostics**
Clinical investigation Laboratory testing incl. tumor markers Computed tomography (CT) of the chest, abdomen and pelvis with oral, rectal and intravenous contrast

**Tumor-specific essential diagnostics**

*CRC: *complete colonoscopy *GC: *gastroscopy

**Useful additional diagnostics (case-dependent)**

Ultrasonography Magnetic resonance imaging (MRI) Positron emission tomography (PET)/PET-CT Diagnostic laparoscopy

CRC: colorectal cancer, GC: gastric cancer

### Morbidity and mortality

In the literature morbidity and mortality rates after CRS and HIPEC range from 25% to 41% and from 0% to 8%, respectively (Table 1) [25-38]. Morbidity can be divided in surgery-related and chemotherapy-related complications. Common surgery-related complications are for example postoperative ileus, anastomotic leakage, wound infection, bleeding, thrombosis and lung embolism. The different cytostatic agents used for HIPEC can lead to leucopenia, anemia, thrombopenia, heart, liver or renal toxicity and other side effects.

In a prospective study of 70 patients with DMPM Yan et al. found primary colonic anastomosis, more than four peritonectomy procedures (total anterior parietal peritonectomy, greater omentectomy/splenectomy, subphrenic peritonectomy, pelvic peritonectomy, lesser omentectomy/cholecystectomy) and operating time greater than 7 hours to be associated with grade IV morbidity [27]. The grade III and IV morbidity rate were 27% and 14%, respectively. The perioperative mortality rate was 3%. Hansson et al. analyzed 123 patients treated with CRS and HIPEC for peritoneal carcinomatosis [39]. The grade III/IV morbidity rate and the treatment-related mortality rate were 41% and 4%, respectively. Bowel morbidity was associated with electroevaporation or excision of tumor nodes on the small bowel surface. In conclusion, morbitity rates after CRS and HIPEC are relatively high but comparable to other major gastrointestinal surgery. However, in the existing studies the assessment of morbidity is not standardized and therefore often not comparable. Thus, following the consensus statement from Milan in further studies the classification system CTCAE version 3.0 should be used. Morbidity is classified in minor complications (grade 0 to 2) and major complications (grade 3 to 5). Moreover, the classification system includes 28 categories leading to an efficient assessment of morbidity [40].

### Survival rates

Several studies have shown that CRS and HIPEC as an integrative part of an interdisciplinary cancer treatment concept may improve survival of patients with peritoneal dissemination of different tumor entities such as colorectal cancer (CRC), gastric cancer (GC), ovarian cancer (OC) and diffuse malignant peritoneal mesothelioma (DMPM) (Table 1).

There are two prospective randomized controlled trials (RCT), one non-randomized comparative study and numerous observational studies regarding clinical and oncologiocal outcome of patients with peritoneal carcinomatosis arising from CRC. Verwaal et al. reported a disease-specific survival of 22.2 months after additional CRS and HIPEC vs. 12.6 months after standard systemic treatment with 5-FU and leucovorin [25,41]. In patients with complete macroscopic cytoreduction (CCR-0/1) median survival was 48 months and 5-year survival rate was 45%, respectively. The second RCT was closed after inclusion of only 35 patients during a 4 year accrual period. The 2-year survival rates were 60% in both arms [42]. In the comparative study published by Mahteme et al. the median survival in the HIPEC group was 32 months vs. 14 months in the control group. 5-year survival rates were 28% and 5% respectively [43]. In the observational studies the overall median survival ranged from 15 to 32 months and from 28 to 60 months after complete macroscopic cytoreduction (CCR0-1), respectively [9].

The prognosis of patients with peritoneal tumor dissemination from GC is poor but could be significantly improved by CRS and HIPEC in selected patients. Six observational studies including between 17 and 154 patients showed median survival rates ranging from 10 to 19 months [28-31,44,45]. The 5-year survival rates after complete macroscopic cytoreduction (CCR-0/1) were 21%, 27%, 29%, 31% and 32%, respectively. Yonemura et al. could show in a multivariate analysis that the completeness of cytoreduction is a highly significant factor for the prediction of patient survival. Moreover, low PCI as well as P1/P2 using the Japanese classification or stage I/II using the Lyon classification indicating limited extent of peritoneal tumor dissemination were associated with better prognosis [46].

Cytoreductive surgery has already been shown to improve survival of patients with stage III and IV ovarian cancer previous to introduction of the combined treatment concept with CRS and HIPEC [10]. Nevertheless, further improvement of long-term survival is reported for CRS and HIPEC in selected patients. In several studies the median survival rates range from 28 to 46 months and 5-year survival rates from 15 to 50% [26].

DMPM is a rare disease with relatively low incidence. Thus, in the systemic review published by Yan et al. survival data of only seven studies including 12 to 100 patients are reported [47]. In these studies median survival ranges between 34 and 92 months and the 5-year survival rates between 33% and 59%, respectively. All studies showed a significant improvement of survival compared to historical controls. Nevertheless, a prospective randomized controlled trial comparing the best available therapy – especially after introduction of permetrexed for the systemic treatment of DMPM – with or without CRS and HIPEC is still not available.

### Quality of life after CRS and HIPEC

Despite relatively high morbidity rates and consecutive initial impairment of quality of life (QoL) several studies could show an improvement of QoL after CRS and HIPEC in long-term survivors [48-52]. McQuellon et al. reported an initial decrease of physical, functional and well-being scores with an increase relative to baseline levels during follow-up at 3, 6 and 12 months. One year after surgery 74% of the patients resumed > 50% of their normal activities [49]. In another publication McQuellon et al. concluded that acceptable QoL, return of functional status and reduced pain can be attained 3 to 6 months after CRS and HIPEC. However, a significant number of patients show depressive symptoms at the time of surgery (32%) as well as one year after surgery (24%) [52]. Schmidt et al. evaluated QoL after CRS and HIPEC in 67 patients with peritoneal carcinomatosis using the EORTC QLQ-C30 questionnaire. The mean score for global health status of long-term survivors was significantly decreased compared to the control population (62.6 vs. 73.3) showing particularly an impairment of role and social functioning [48]. Tuttle et al. showed a return of QoL measurements to baseline 4 months after surgery in a prospective analysis of 35 patients. Eight and twelve months after CRS and HIPEC QoL was significantly improved [51]. In conclusion, the existing studies show that CRS and HIPEC can be performed with acceptable postoperative QoL and even may improve QoL in a selected part of long-term survivors.

## Conclusion

Cytoreductive surgery and hyperthermic intraperitoneal chemotherapy provide a promising therapeutic option for highly selected patients with peritoneal carcinomatosis arising from different malignancies such as colorectal cancer, gastric cancer, ovarian cancer or peritoneal mesothelioma. Numerous studies with different levels of evidence have shown that the integration of CRS and HIPEC in an interdisciplinary treatment concept may improve the oncological outcome compared to sole palliative systemic chemotherapy. The completeness of cytoreduction plays a pivotal role for long-term survival. Thus, consequent preoperative diagnostic workup and patient selection is essential. The existing studies also show that the combined treatment concept can be performed with low mortality and acceptable morbidity in specialized centers. The rate of complications is influenced by the extent of surgery and the cytostatic agent used for intraperitoneal application and its concentration. The quality of life is initially impaired by surgery and postoperative complications. Nevertheless, the functional status returns to baseline in most patients during the first 4 moths after surgery. In selected patients QoL may even be improved one year or later after surgery.

However, for most tumor entities prospective randomized controlled trials comparing best available therapy using new therapeutic agents and combined systemic chemotherapy with and without CRS and HIPEC are still not available. Such studies may provide higher levels of evidence in the future and help to determine the significance of CRS and HIPEC as an integrative part of an interdisciplinary cancer treatment strategy in selected patients with peritoneal carcinomatosis.

## Competing interests

The authors declare that they have no competing interests.

## Authors' contributions

GG drafted the manuscript. HJS corrected the manuscript. PP drafted and corrected the manuscript. All authors read and approved the final manuscript.
